# Next-generation sequencing of 500 POI patients identified novel responsible monogenic and oligogenic variants

**DOI:** 10.1186/s13048-023-01104-6

**Published:** 2023-02-15

**Authors:** Wei Luo, Hanni Ke, Shuyan Tang, Xue Jiao, Zhuqing Li, Shidou Zhao, Feng Zhang, Ting Guo, Yingying Qin

**Affiliations:** 1 Center for Reproductive Medicine, Shandong University. Department of Obstetrics and Gynecology, Shandong Provincial Hospital Affiliated to Shandong First Medical University, Jinan Shandong.;Shandong Provincial Hospital. National Research Center for Assisted Reproductive Technology and Reproductive Genetics, China. Key laboratory of Reproductive Endocrinology of Ministry of Education, Shandong Provincial Clinical Medicine Research Center for Reproductive Health, Jinan, China; 2 Center for Reproductive Medicine, Shandong University. National Research Center for Assisted Reproductive Technology and Reproductive Genetics, China. Key laboratory of Reproductive Endocrinology of Ministry of Education, Shandong Provincial Clinical Medicine Research Center for Reproductive Health, Shandong University, Jinan, China; 3 Obstetrics and Gynecology Hospital, School of Life Sciences, Fudan University, Shanghai, China

**Keywords:** Premature ovarian insufficiency, Next generation sequencing, Targeted gene panel, Mutation

## Abstract

**Background:**

Premature ovarian insufficiency refers to the loss of ovarian function before 40 years of age. The etiology is heterogeneous, and genetic factors account for 20–25% of cases. However, how to transform genetic findings to clinical molecular diagnose remains a challenge. To identify potential causative variations for POI, a next generation sequencing panel with 28 known causative genes of POI was designed, and a large cohort of 500 Chinese Han patients was screened directly. Pathogenic evaluation of the identified variants and the phenotype analysis were performed according to monogenic or oligogenic variants.

**Results:**

A total of 14.4% (72/500) of the patients carried 61 pathogenic or likely pathogenic variants in 19 of the genes in the panel. Interestingly, 58 variants (95.1%, 58/61) were firstly identified in patients with POI. FOXL2 harbored the highest occurrence frequency (3.2%, 16/500), among whom presented with isolated ovarian insufficiency instead of blepharophimosis-ptosis-epicanthus inversus syndrome. Moreover, luciferase reporter assay confirmed variant p.R349G, which account for 2.6% of POI cases, impaired the transcriptional repressive effect of FOXL2 on *CYP17A1*. The novel compound heterozygous variants in *NOBOX* and *MSH4* were confirmed by pedigree haplotype analysis, and digenic heterozygous variants in *MSH4* and *MSH5* were firstly identified. Furthermore, nine patients (1.8%, 9/500) with digenic or multigenic pathogenic variants presented with delayed menarche, early onset of POI and high prevalence of primary amenorrhea compared with those with monogenic variation(s).

**Conclusions:**

The genetic architecture of POI has been enriched through the targeted gene panel in a large cohort of patients with POI. Specific variants in pleiotropic genes may result in isolated POI rather than syndromic POI, whereas oligogenic defects might have cumulative deleterious effects on the severity of POI phenotype.

## Background

Premature ovarian insufficiency (POI) is defined as loss of ovarian function before 40 years of age, and it is characterized by oligomenorrhea or amenorrhea, elevated follicle stimulating hormone (FSH), and decreased estrogen. Approximately 1%–5% of women under 40 years old are diagnosed with POI, and present with infertility, estrogen deficiency symptoms, and long-term complications, such as osteoporosis and cardiovascular disease [1]. The etiology of POI is heterogeneous, including genetic factors, autoimmune disorders, infections, and iatrogenic causes [2]. Genetic factors account for 20%–25% of cases [3], including chromosome abnormalities and gene mutations. However, high genetic heterogeneity exists in both isolated and syndromic patients. Currently, nearly 80 genes have been reported to be associated with POI, while only a small subset of these genes can explain more than 5% of patients [3, 4]. Due to limited gene coverage, high time consumption and expense, monogenic screening by Sanger sequencing has seldom been used in genetic studies of POI.

Recently, with the application of whole exome sequencing (WES) in POI pedigrees, vast numbers of genetic variants have been emerged in the omics era. Especially, based on the expanded genetic spectrum of POI, next-generation sequencing (NGS) of POI genes makes its genetic diagnosis possible. Fonseca et al. performed NGS covering 70 candidate genes in 12 patients with POI and found 25% of the patients carried potential mutations [5]. Bouilly et al. sequenced 19 candidate genes using NGS in 100 patients and identified a mutation frequency of 19% [6]. A higher frequency of mutation carrier (48%) has been reported in 69 POI patients through NGS of 420 candidate genes [7]. More recently, Raffaella et al. developed a 295 genes panel and screened in 64 patients, founding that the phenotypes of POI were associated with the number of variations, which supports the oligogenic nature of POI [8]. However, majority of candidate genes designed in above-mentioned panels were selected based on the phenotypes of animal models or related biological functions, lacking solid evidence in human POI. It is impractical for molecular diagnosis of POI in the absence of sufficient functional evidence. Therefore, how to improve the diagnostic efficacy of gene panel is still challenging for POI patients.

Here, we designed a targeted gene panel consisting of 28 known causative genes of human POI. A large cohort of 500 POI patients were enrolled and screened with the panel to expand the genetic spectrum and architecture of POI.

## Results

### Genetic analysis

**Fig. 1:**
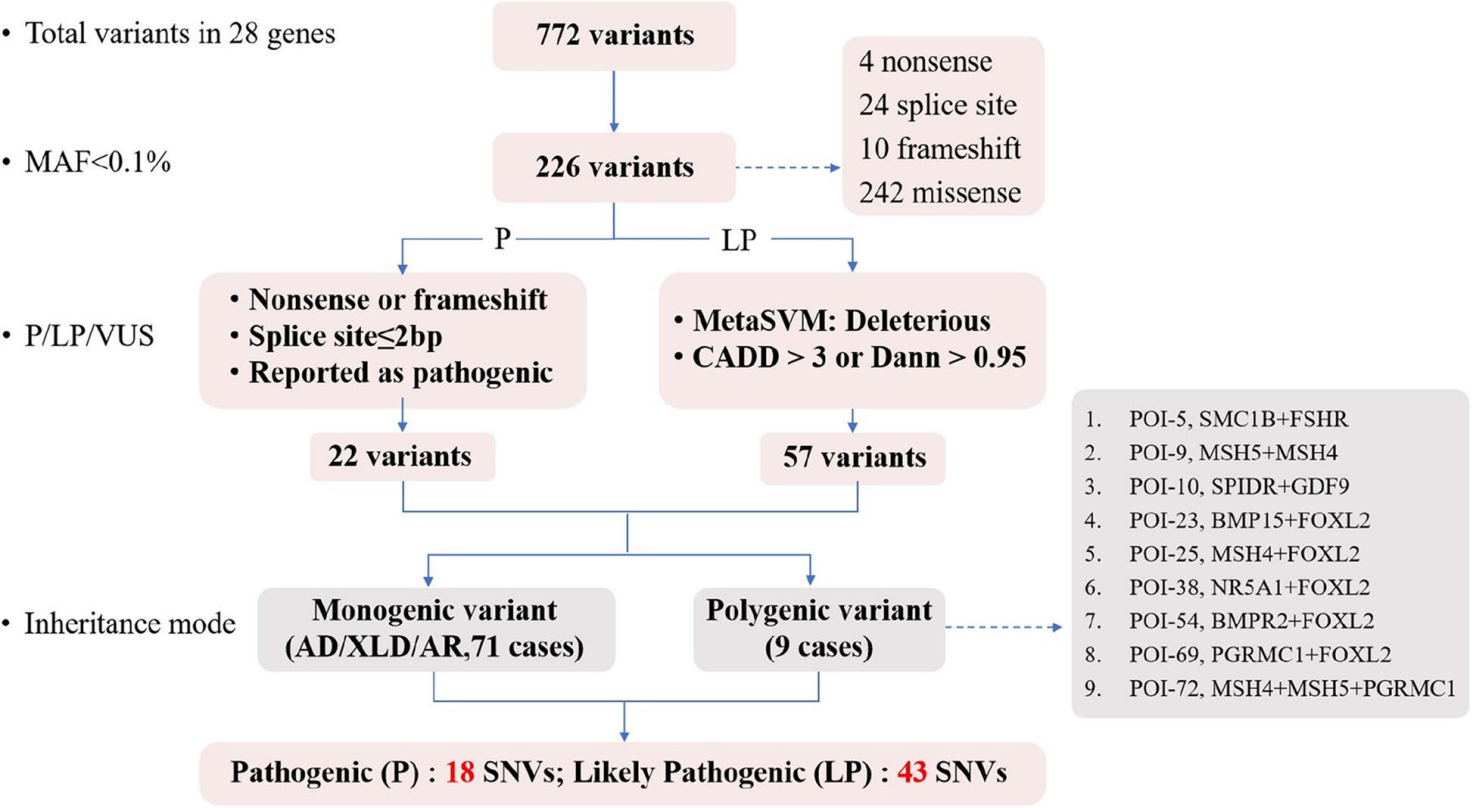
Overview of the filtering process of potential causative variants for POI. The genetic panel included 28 POI candidate genes. A total of 772 variants were identified by panel testing, of which 226 variants had rare allele frequencies (< 0.1%), including 22 pathogenic (P) variants and 57 likely pathogenic [9] variants. Among the patients carrying P or LP variants, 71 cases could be explained by monogenic variants and 9 cases had oligogenic variants located in more than one gene. Finally, 61 variants (18 pathogenic and 43 likely pathogenic) were filtered and confirmed by Sanger sequencing. MAF: minor allele frequency; P: pathogenic; LP: likely pathogenic; VUS: variant of uncertain significance; CADD: combined annotation dependent depletion; DANN: deleterious annotation of genetic variants using neural networks; SNV: single-nucleotide variant

In the 500 Chinese Han patients with POI, a total of 772 sequence variants were identified in the 28 genes. Among them, 226 rare variants (frequency < 0.1% in the 1000 Genomes Project and gnomAD databases) were further analyzed, including 4 nonsense, 24 splice site, 10 frameshift, and 242 missense variants. After filtering by MetaSVM, CADD, and DANN score, 22 pathogenic and 57 likely pathogenic variants were considered as potentially causative for POI (Fig. 1).

**Fig. 2:**
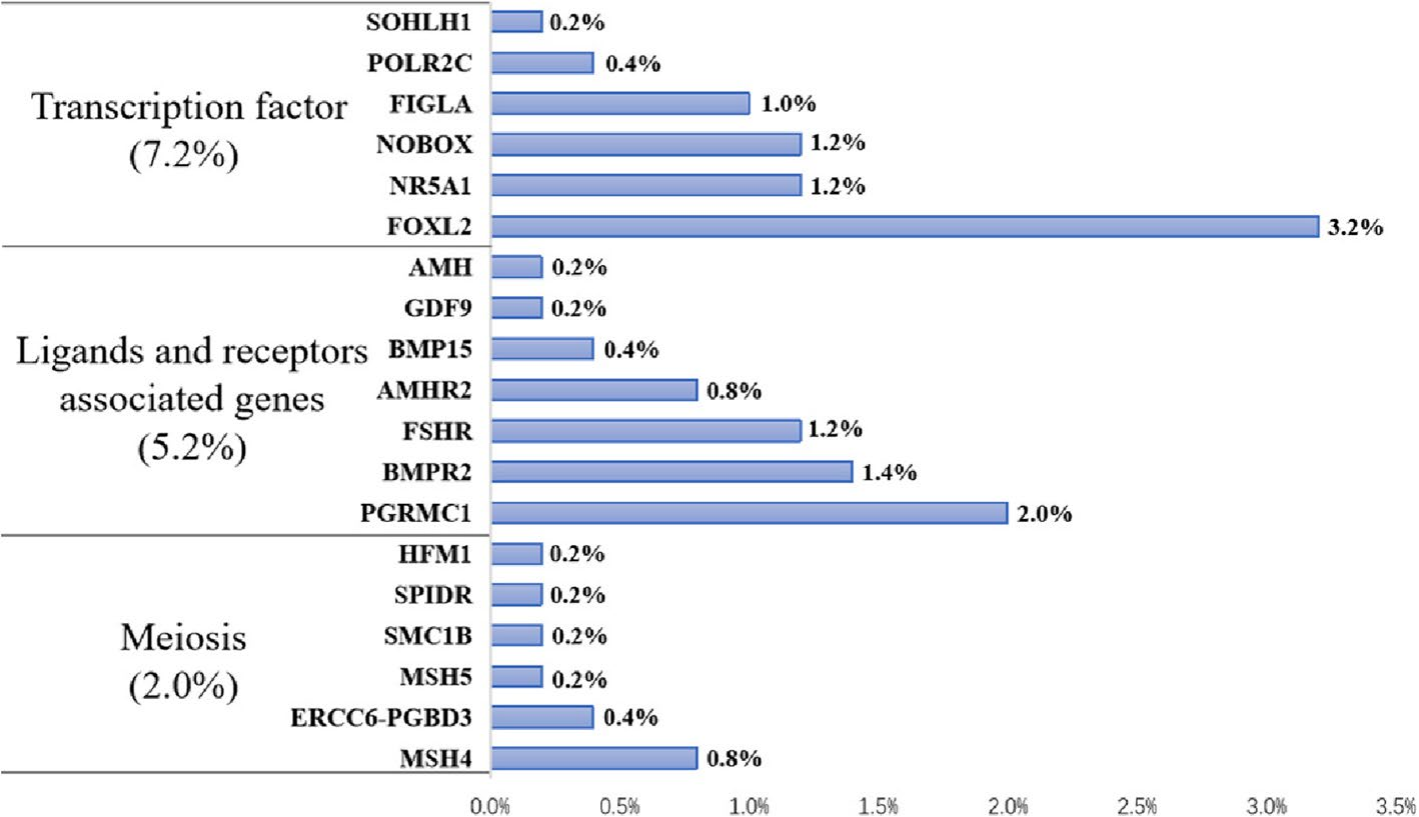
Prevalence of candidate gene variants identified in POI patients. The 61 pathogenic/likely pathogenic filtered variants were located in 19 genes, including 6 meiosis genes, 6 transcription factors, and 7 genes involved in steroid hormone synthesis or response pathways. Among these, the transcription factors had the highest mutation frequency (7.2%), followed by steroid hormone synthesis or receptor genes (6.2%) and meiotic genes (2.0%)

According to classical inheritance patterns (autosomal recessive or dominant, X-linked recessive or dominant patterns), 71 patients could be explained by monogenic variants, and 8 of them carried additional P/LP variants located in one or two other genes. Intriguingly, in addition to these 71 patients, one patient carried digenic heterozygous variants in *MSH4* and *MSH5,* which interacted to form a heterodimer during homologous recombination and were thought to be causative for POI in recessive pattern separately. Therefore, 72 patients carried pathogenic or likely pathogenic variants were screened out, and 9 of them had digenic or multigenic variants (Fig. 1). A total of 18 pathogenic and 43 likely pathogenic variants located in 19 genes (Table 1, Fig. 2), i.e. 6 meiosis genes (*HFM1*, *SPIDR*, *SMC1B*, *MSH5*, *MSH4*, *CSB-PGBD3*), 6 transcription factors (*SOHLH1*, *POLR2C*, *FIGLA*, *NOBOX*, *NR5A1*, *FOXL2*) and 7 genes involved in ligands and receptors (*AMH*, *AMHR2*, *GDF9*, *BMP15*, *FSHR*, *BMPR2*, *PGRMC1*). Interestingly, only three of the variants had been reported in previous studies (p.R355H in *NOBOX*, p.R313H in *NR5A1*, and p.R351G in *MSH5*) [10–13], whereas the other 58 variants were firstly identified in human POI (Table 1).

**Table 1:** Clinical characteristics and molecular findings of POI patients carrying pathogenic or likely pathogenic variants

**Patients ID**	**Phenotype**^a^	**Menarche age**	**POI onset age**	**Hormone values**^b^			**Gene**	**Accession number**	**Mutation**		**SNP ID**	**Classification**^d^	**Allele frequency**	
				**FSH (IU/L)**	**LH (IU/L)**	**E2 (ng/L)**			**Sequence variation**	**Protein position**			**GnomAD**	**ExAC_EAS**
POI-1	SA	15	26	73.35	41.08	5.60	*NOBOX*	NM_001080413.3	c.2041dupG	p.A681fs		P	1.46E-05	
POI-2	SA	15	15	109.90	57.32	< 5.00	*FOXL2*	NM_023067.4	c.1045C > G	p.R349G	rs201840174	LP	3.14E-04	2.40E-03
POI-3	SA	12	31	39.91	36.25	80.60	*FOXL2*	NM_023067.4	c.1045C > G	p.R349G	rs201840174	LP	3.14E-04	2.40E-03
POI-4	SA	13	17	101.80	40.84	< 11.80	*BMPR2*	NM_001204.7	c.1042G > A	p.V348I	rs201067849	LP	5.23E-04	6.40E-03
POI-5 ^#^	PA	19	19	43.56	40.01	16.40	*SMC1B*	NM_148674.5	c.863A > G	p.E288G	rs781614640	LP	1.30E-05	2.00E-04
							*FSHR*	NM_000145.4	c.884C > T	p.S295F		LP		
							*FSHR*	NM_000145.4	c.374 T > G	p.L125R		LP		
POI-6	SA	15	28	69.89	32.20	73.21	*MSH4*	NM_002440.4	c.2220_2223del	p.M740fs		P	4.77E-06	
							*MSH4*	NM_002440.4	c.2728C > T	p.R910X	rs771456188	P	8.71E-06	
POI-7	SA	11	20	74.41	30.80	2.12	*FIGLA*	NM_001004311.3	c.11C > A	p.A4E	rs71647803	LP		
POI-8	SA	12	25	81.03	55.21	11.40	*FOXL2*	NM_023067.4	c.789C > A	p.S263R		LP		
POI-9 ^#^	SA	15	20	46.08	32.33	80.67	*MSH5*	NM_002441.4	c.826C > T	p.R276C	rs144471639	LP	4.07E-06	
							*MSH4*	NM_002440.4	c.1063A > G	p.I355V	rs116141807	LP	1.71E-04	2.90E-03
POI-10 ^#^	SA	14	24	62.40	11.80	< 20.00	*SPIDR*	NM_001282916.1	c.566 + 1G > T	-	rs781987881	P	4.15E-06	1.00E-04
							*SPIDR*	NM_001282916.1	c.2054delC	p.S685fs	rs754810654	P	3.25E-05	6.00E-04
							*GDF9*	NM_005260.5	c.1283G > C	p.S428T	rs118080183	LP	2.34E-04	4.00E-03
POI-11	SA	15	ND	54.61	66.21	13.50	*MSH4*	NM_002440.4	c.2220_2223del	p.M740fs		P	4.77E-06	
							*MSH4*	NM_002440.4	c.2374A > G	p.T792A	rs557796016	LP		5.00E-04
POI-12	SA	13	28	40.59	27.66	35.71	*FSHR*	NM_000145.4	c.374 T > G	p.L125R		LP		
POI-13	SA	13	23	18.11	6.22	11.43	*BMPR2*	NM_001204.7	c.1585C > T	p.R529C	rs140049204	LP	4.87E-05	
POI-14	SA	14	16	71.56	45.34	42.70	*BMPR2*	NM_001204.7	c.2618G > A	p.R873Q	rs201781338	LP	9.74E-05	1.50E-03
POI-15	PA	16	23	59.93	18.96	7.20	*FIGLA*	NM_001004311.3	c.319C > T	p.L107F		LP	4.17E-06	
POI-16	SA	13	26	65.71	42.52	18.80	*FIGLA*	NM_001004311.3	c.11C > A	p.A4E	rs71647803	LP		
POI-17	PA	18	18	131.1	58.19	< 5.00	*AMHR2*	NM_020547.3	c.775C > T	p.R259X	rs746905091	P	4.06E-06	1.00E-04
POI-18	SA	12	18	77.54	36.67	< 5.00	*AMHR2*	NM_020547.3	c.515G > A	p.R172Q		LP	4.10E-06	
POI-19	SA	14	14	24.89	8.18	51.20	*FOXL2*	NM_023067.4	c.1045C > G	p.R349G	rs201840174	LP	3.14E-04	2.40E-03
POI-20	SA	13	20	77.32	38.22	< 5.00	*GDF9*	NM_005260.5	c.946C > T	p.R316C	rs751894227	LP	8.12E-06	
POI-21	SA	12	27	92.73	56.51	52.20	*POLR2C*	NM_032940.3	c.544G > T	p.V182L		LP		
POI-22	PA	16	20	ND	ND	ND	*HFM1*	NM_001017975.6	c.1978-2A > C	-		P		
							*HFM1*	NM_001017975.6	c.1880 T > C	p.V627A		LP	4.10E-06	
POI-23 ^#^	SA	15	17	107.78	29.87	24.08	*BMP15*	NM_005448.2	c.208A > C	p.M70L	rs782325962	LP	7.05E-06	5.00E-04
							*FOXL2*	NM_023067.4	c.1045C > G	p.R349G	rs201840174	LP	3.14E-04	2.40E-03
POI-24	SA	14	30	33.59	17.86	103.77	*NR5A1*	NM_004959.5	c.250C > T	p.R84C		LP		
POI-25 ^#^	PA	17	17	36.98	10.56	5.30	*MSH4*	NM_002440.4	c.1855A > G	p.M619V		LP	0.00E + 00	
							*FOXL2*	NM_023067.4	c.1045C > G	p.R349G	rs201840174	LP	3.14E-04	2.40E-03
POI-26	SA	14	30	76.15	57.85	12.90	*NOBOX*	NM_001080413.3	c.1298G > T	p.G433V		LP	4.31E-06	
POI-27	SA	15	31	134.50	64.46	32.43	*SOHLH1*	NM_001101677.2	c.244C > G	p.Q82E		LP		
POI-28	SA	14	30	90.65	59.31	< 5.00	*BMPR2*	NM_001204.7	c.1481C > T	p.A494V	rs2229778	LP	6.13E-05	6.00E-04
POI-29	SA	12	32	71.96	48.51	21.53	*NR5A1*	NM_004959.5	c.565C > A	p.P189T		LP		
POI-30	SA	13	23	113.67	31.46	5.79	*PGRMC1*	NM_006667.5	c.418G > C	p.D140H		LP		
POI-31	SA	12	27	ND	ND	ND	*ERCC6-PGBD3*	NM_001277059.2	c.814G > A	p.E272K	rs768589918	LP	8.12E-06	
POI-32	SA	12	26	ND	ND	ND	*PGRMC1*	NM_006667.5	c.533C > T	p.T178I	rs201254642	LP	2.55E-04	3.50E-03
POI-33	SA	13	24	ND	ND	ND	*PGRMC1*	NM_006667.5	c.272 T > C	p.M91T	rs776947628	LP	3.86E-05	2.00E-04
POI-34	SA	14	35	ND	ND	ND	*FOXL2*	NM_023067.4	c.1045C > G	p.R349G	rs201840174	LP	3.14E-04	2.40E-03
POI-35	SA	15	ND	ND	ND	ND	*FSHR*	NM_000145.4	c.1396G > A	p.E466K		LP		
POI-36	SA	14	ND	ND	ND	ND	*PGRMC1*	NM_006667.5	c.533C > T	p.T178I	rs201254642	LP	2.55E-04	3.50E-03
POI-37	SA	13	24	125.53	57.32	< 5.00	*FOXL2*	NM_023067.4	c.1045C > G	p.R349G	rs201840174	LP	3.14E-04	2.40E-03
POI-38 ^#^	PA	16	24	59.18	28.09	40.32	*NR5A1*	NM_004959.5	c.559delG	p.A187fs		P		
							*FOXL2*	NM_023067.4	c.1045C > G	p.R349G	rs201840174	LP	3.14E-04	2.40E-03
POI-39	SA	ND	ND	49.22	21.95	17.64	*FOXL2*	NM_023067.4	c.553G > C	p.G185R		LP		
POI-40	SA	13	37	58.16	18.42	20.24	*FSHR*	NM_000145.4	c.349C > T	p.Q117X		P		
POI-41	SA	14	14	60.82	9.75	< 20.00	*NR5A1*	NM_004959.5	c.909C > A	p.S303R		LP		
POI-42	SA	14	20	121.53	110.57	20.53	*BMPR2*	NM_001204.7	c.1042G > A	p.V348I	rs201067849	LP	5.23E-04	6.40E-03
POI-43	SA	14	31	60.13	37.06	5.20	*NOBOX*	NM_001080413.3	c.1131delT	p.P377fs		P		
POI-44	PA	16	30	36.21	40.54	88.54	*PGRMC1*	NM_006667.5	c.533C > T	p.T178I	rs201254642	LP	2.55E-04	3.50E-03
POI-45	SA	13	25	60.49	39.16	48.68	*ERCC6-PGBD3*	NM_001277059.2	c.3100G > T	p.E1034X	rs866374385	P	3.23E-05	
POI-46	SA	10	26	77.32	44.05	14.32	*PGRMC1*	NM_006667.5	c.533C > T	p.T178I	rs201254642	LP	2.55E-04	3.50E-03
POI-47	PA	17	20	76.23	22.59	39.64	*FOXL2*	NM_023067.4	c.871dupC	p.H291fs		P		
POI-48	SA	ND	ND	ND	ND	ND	*POLR2C*	NM_032940.3	c.77C > G	p.T26S	rs770336099	LP	8.28E-05	1.30E-03
POI-49	SA	13	35	91.58	37.34	10.35	*MSH5*	NM_002441.4	c.1051C > G	p.R351G	rs28399976	P		
POI-50	PA	16	16	46.34	9.54	< 5.00	*NOBOX*	NM_001080413.3	c.1199C > T	p.P400L	rs568492478	LP	5.92E-05	2.70E-03
POI-51	PA	17	17	ND	ND	ND	*NR5A1*	NM_004959.5	c.937C > T	p.R313C		LP		
POI-52	SA	14	25	59.32	17.78	12.32	*NOBOX*	NM_001080413.3	c.1674_1677del	p.L558fs		P		
							*NOBOX*	NM_001080413.3	c.1064G > A	p.R355H	rs201947677	P	1.59E-04	
POI-53	PA	19	19	ND	ND	ND	*AMHR2*	NM_020547.3	c.55C > G	p.P19A	rs756301317	LP	5.28E-05	1.50E-03
POI-54 ^#^	PA	17	17	83.46	39.56	9.20	*FOXL2*	NM_023067.4	c.1045C > G	p.R349G	rs201840174	LP	3.14E-04	2.40E-03
							*BMPR2*	NM_001204.7	c.1042G > A	p.V348I	rs201067849	LP	5.23E-04	6.40E-03
POI-55	SA	13	19	79.35	44.95	3.99	*AMH*	NM_000479.5	c.1393C > T	p.R465C		LP		
POI-56	PA	16	16	75.53	24.05	5.50	*AMHR2*	NM_020547.3	c.355A > G	p.N119D		LP		
POI-57	SA	12	20	91.66	41.98	14.20	*PGRMC1*	NM_006667.5	c.533C > T	p.T178I	rs201254642	LP	2.55E-04	3.50E-03
POI-58	SA	14	31	66.95	37.22	24.43	*BMP15*	NM_005448.2	c.919C > T	p.H307Y	rs782540417	LP	1.23E-05	3.00E-04
POI-59	SA	15	37	55.49	40.04	59.33	*FOXL2*	NM_023067.4	c.1045C > G	p.R349G	rs201840174	LP	3.14E-04	2.40E-03
POI-60	SA	13	ND	40.65	20.09	< 10.00	*FIGLA*	NM_001004311	c.326G > A	p.G109D		LP		
POI-61	SA	15	ND	96.01	60.93	71.34	*FSHR*	NM_001004311.3	c.1763 T > C	p.I588T		LP		
							*FSHR*	NM_000145.4	c.683C > T	p.T228I	rs776897994	LP	4.08E-06	0.00E + 00
POI-62	PA	18	18	51.89	33.83	26.55	*FSHR*	NM_000145.4	c.1679_1685del	p.N560fs		P		
POI-63	PA	19	26	117.83	41.01	7.83	*MSH4*	NM_000145.4	c.2220_2223del	p.M740fs		P	4.77E-06	
							*MSH4*	NM_002440.4	c.2374A > G	p.T792A	rs557796016	LP		5.00E-04
POI-64	SA	13	22	104.93	33.36	59.22	*FIGLA*	NM_002440.4	c.11C > A	p.A4E	rs71647803	LP		
POI-65	SA	14	25	107.22	57.03	18.35	*FOXL2*	NM_001004311.3	c.1045C > G	p.R349G	rs201840174	LP	3.14E-04	2.40E-03
POI-66	SA	15	28	65.96	22.86	35.15	*NR5A1*	NM_023067.4	c.938G > A	p.R313H	rs1554721235	P		
POI-67	PA	16	16	54.97	16.16	19.32	*FOXL2*	NM_023067.4	c.1045C > G	p.R349G	rs201840174	LP	3.14E-04	2.40E-03
POI-68	SA	15	18	124.66	47.81	16.21	*BMPR2*	NM_001204.7	c.960_961insCGA	p.P320fs		P		
POI-69 ^#^	PA	16	26	91.68	58.68	< 5.00	*PGRMC1*	NM_006667.5	c.533C > T	p.T178I	rs201254642	LP	2.55E-04	3.50E-03
							*FOXL2*	NM_023067.4	c.1045C > G	p.R349G	rs201840174	LP	3.14E-04	2.40E-03
POI-70	SA	15	21	98.26	36.23	23.13	*NOBOX*	NM_001080413.3	c.1199C > T	p.P400L	rs568492478	LP	5.92E-05	2.70E-03
POI-71	SA	15	27	141.95	57.14	< 5.00	*PGRMC1*	NM_006667.5	c.533C > T	p.T178I	rs201254642	LP	2.55E-04	3.50E-03
POI-72 ^#^	SA	15	ND	41.11	24.45	< 5.00	*MSH5*	NM_002441.4	c.1051C > G	p.R351G	rs28399976	P		
							*MSH4*	NM_002440.4	c.1025C > T	p.T342I	rs777079867	LP	4.72E-06	2.00E-04
							*MSH4*	NM_002440.4	c.2220_2223del	p.M740fs		P	4.77E-06	
							*PGRMC1*	NM_006667.5	c.533C > T	p.T178I	rs201254642	LP	2.55E-04	3.50E-03

^a^*PA* Primary amenorrhea, *SA* Secondary amenorrhea

^b^ The clinical features of some patients are not determined (ND)

^c^ The splicing region mutations are marked as “-”; the frameshift mutations are marked as “fs”

^d^ The classification of variation: pathogenic as “P”; likely pathogenic as “LP”

^#^ The patients with digenic or multigenic pathogenic variants

**Fig. 3:**
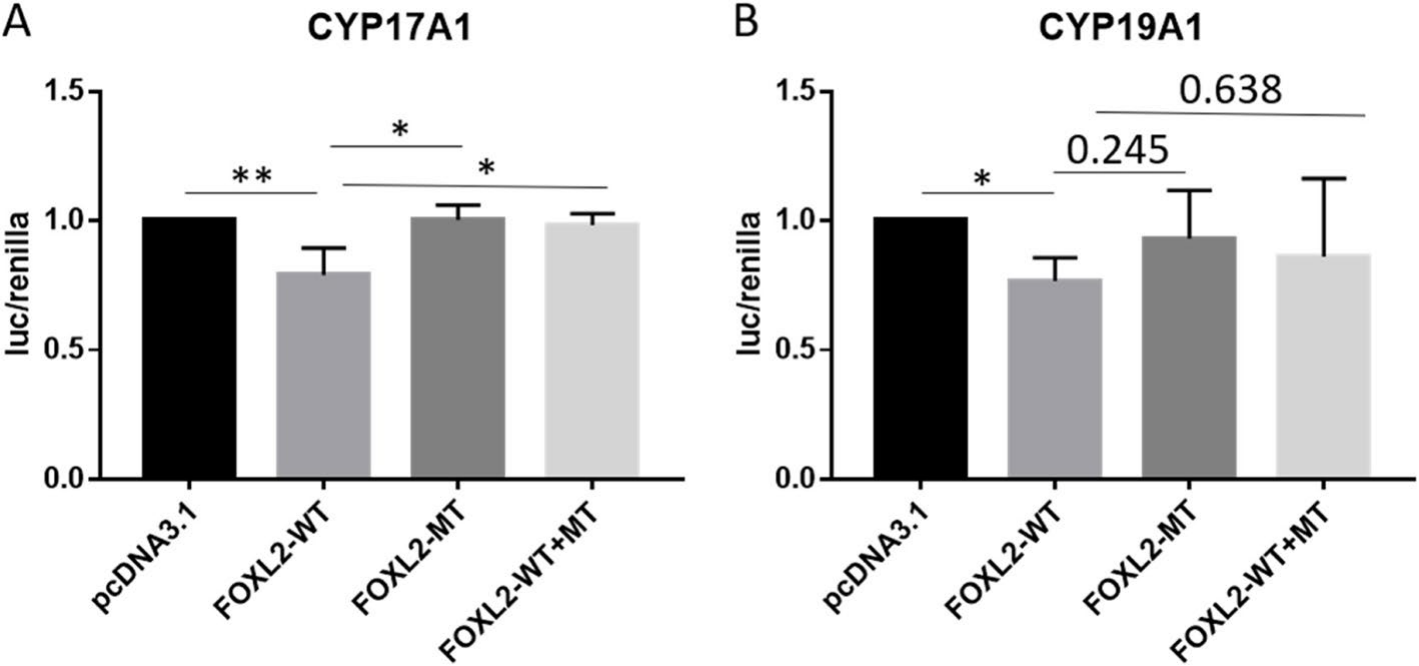
Variant p.R349G affected the transcriptional effect of FOXL2. The pcDNA3.1 vector, wild-type (WT) or/and mutant (MT) FOXL2 vectors and CYP17A1 (**A**)/CYP19A1 (**B**) promoter reporter were co-transfected in HEK392 cells. The results are shown as luciferase/renilla signal, and the pcDNA3.1 group was used as the control. The potential dominant-negative effect of FOXL2 p.R349G mutation was assessed by co-transfecting WT and MT vectors in 1:1 ratio. * indicated the *p*-value < 0.05, ** indicated the *p*-value < 0.01

Among the 28 genes, *FOXL2* harbored the highest variant occurrence frequency. Sixteen patients carried *FOXL2* heterozygous variants (16/500, 3.20%), 13 of whom with variant c.1045C > G (p.R349G) (13/500, 2.60%). The frequency of p.R349G in POI patients was significantly higher than that in 1000 Genomes database (0.08%, *p* = 0.001) and East Asians of the ExAC database (0.24%, *p* < 0.001). To determine whether p.R349G influenced the transcriptional effect of FOXL2, the luciferase reporter assay was performed. The results showed that wild-type *FOXL2* down-regulated the expression of *CYP17A1*, while the mutant *FOXL2* did not present with the transcriptional repressive effect. For the transcriptional regulation of *CYP19A1*, wild-type *FOXL2* showed similar transcriptional repression, whereas, the adverse effect of mutant *FOXL2* was not significant (Fig. 3).

### Pedigree analysis

**Fig. 4:**
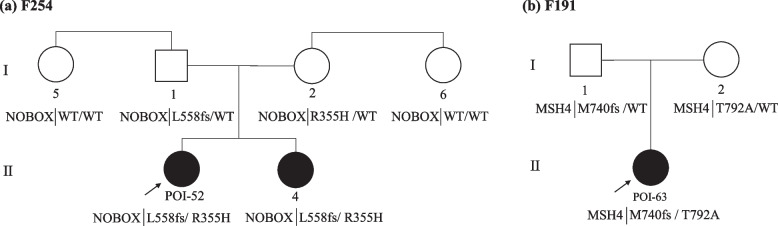
POI pedigrees harboring compound heterozygous variants or multigenic variants. The probands are marked with arrows, women with POI are depicted by black circles, and healthy women are depicted by white circles and healthy men by white squares. **a** In pedigree F254, the proband and her sister carried the compound heterozygous variation *NOBOX* p.L558fs and p.R355H, which was inherited from their father and mother separately. **b** In pedigree F191, proband POI-63 carried compound heterozygous mutation *MSH4* p.M740fs and p.T792A, which was inherited from her father and mother, respectively.

Compound heterozygous variants in *NOBOX* and *MSH4* were identified, and haplotype analysis was further performed in the POI pedigree with two POI sisters carrying *NOBOX* variants and in one trio family with *MSH4* variants (Fig. 4), respectively.

In pedigree F254 (Fig. 4a), the proband POI-52 and her younger sister carried the compound heterozygous variant p.L558fs and p.R355H in *NOBOX*, which was inherited from their father and mother separately. The proband had menarche at 14 years of age and suffered oligomenorrhea until 25 years of age when she was diagnosed with POI. Her younger sister had secondary amenorrhea at 23 years old and was diagnosed with POI as well. A compound heterozygous variant in *MSH4* (p.M740fs and p.T792A) was identified in POI-63, who had spontaneous menarche at 19 years of age and diagnosed with POI at 31 years old. The compound heterozygous variant was confirmed to be inherited paternally and maternally, respectively (Fig. 4b).

### Clinical characteristics of patients carrying P or LP variants

Among the patients carrying digenic or multigenic variants, a higher percentage of primary amenorrhea (44.44% vs. 19.05%), earlier onset of POI (20.10 ± 6.81 years vs. 24.97 ± 4.67 years), and later menarche (15.82 ± 1.50 years vs. 13.95 ± 2.56 years) were observed compared with women carrying monogenic variants. However, these differences did not reach statistical significance (*p* > 0.05).

## Discussion

In the present study, using a self-designed target panel covering 28 known POI causative genes screened in 500 Chinese Han patients, 14.40% (72/500) of patients were diagnosed with at least one pathogenic variant contributing to ovarian insufficiency. A total of 58 potential causative variants, including digenic heterozygous variants in *MSH4* and *MSH5,* were firstly reported in POI, which not only expanded the variant spectrum of human POI, but also enriched the genetic architecture of POI pathogenesis.

### Variants in pleiotropic genes result in isolated POI

Under most circumstances, the pleiotropic genes responsible for POI cause syndromic POI, which manifests with highly variable somatic abnormalities in addition to reproductive phenotypes, such as *BLM* for Bloom syndrome and *WRN* for Werner syndrome [3]. Recent genetic studies have revealed that variants in pleiotropic genes also resulted in isolated POI, such as *NBN* and *EIF2B2*, which could be explained by specific mutation sites and different types of variants [14]*.* In the present study, variants in pleiotropic genes, including *FOXL2*, *NR5A1*, and *BMPR2*, were identified in patients presenting with isolated POI, confirming that specific variants might contribute to distinct phenotypes of POI. These findings also highlighted the necessity of individualized genetic counseling and long-term healthcare follow-up in these women.

*FOXL2* is preferentially expressed in the ovary, eyelids, and pituitary gland [15, 16]. Heterozygous intragenic variants of *FOXL2* accounted for 71% of patients with blepharophimosis-ptosis-epicanthus inversus syndrome (BPES), which is a dominant condition characterized by eyelid and mild craniofacial defects associated with POI (type I) or not (type II) [17]. Although more than 100 variants of *FOXL2* have been found in BPES patients [17], the constitutional variants were reported in only 1.0%–2.9% of non-syndromic POI cases [18]. Through the panel test, we found that the prevalence of *FOXL2* variants in isolated POI was 3.2%, which was much higher than the other genes in the panel. Intriguingly, the variant p.R349G in *FOXL2* was firstly reported here and accounted for 2.6% of the cohort, which was significantly higher than the frequency in public databases. *FOXL2* is involved in ovarian development by regulating the transcription of essential genes involved in steroidogenesis, including *CYP17A1* and *CYP19A1* [19–22]. Functional studies demonstrated that variant p.R349G impaired the transcriptional regressive effect of FOXL2 on *CYP17A1*, which might further influence the synthesis of estradiol and lead to folliculogenesis abnormalities [21]. Recently, somatic mutation p.C134W in *FOXL2* has been found associated with GCs tumor in adult and accounted for up to 5% of ovarian malignancies [23]. Therefore, although none of the *FOXL2* variation carriers in our cohort presented with eyelid malformation or ovarian tumor, the long-term follow-up is still warranted.

*NR5A1* is a nuclear receptor that regulates the transcription of genes required for adrenal and reproductive development [24]. Variants in *NR5A1* are associated with different reproductive phenotypes in humans, such as disorders of sex development (DSD), hypospadias, and POI. It has been reported that 0.3%–2.3% of POI patients carried *NR5A1* mutations [25, 26], which is similar to the frequency in our study (1.2%). Interestingly, the variation p.R313C, locating at ligand-binding domain of NR5A1*,* was one of the most common variants identified in DSD patients [13]. However, the carriers of p.R313C and p.R313H in our study had normal female external genitalia, which might be explained by genetic heterogeneity during gonad differentiation.

BMPR2 is one of bone morphogenetic protein (BMP) binding soluble factors, participating in signal transduction between oocytes and GCs, which is essential for oocyte maturation [27, 28]. Most *BMPR2* variants were reported previously in patients with idiopathic pulmonary arterial hypertension (IPAH), while recent NGS and functional studies have revealed that p.S987F in *BMPR2* caused isolated POI by perturbing BMP15/BMPR2/SMAD signaling and GCs proliferation [29]. In this study, five variants in *BMPR2* were found for the first time in seven patients with POI. It was reported that the majority of the *BMPR2* variants identified in POI patients were located in cytoplasmic tail (amino acids 504–1038) of BMPR2 [30, 31]. However, three out of the five variants identified here were localized in kinase domain (amino acids 203–503). Similarly, a recent study also identified a novel heterozygous variant of POI patient in BMPR2 kinase domain (p.Val453Met) [32], suggesting that variants located in different domains of *BMPR2* may have individual effects on ovarian function, highlighting the contribution of BMP signal pathway in isolated POI pathogenesis. Although no clinical features of pulmonary hypertension had been found in the time of investigation, the long-term status should be followed.

### Both heterozygous and homozygous variants are pathogenic for POI

The inheritance pattern of POI includes recessive, dominant, and X-linked modes. With the accumulation of variants identified by WES and NGS, more complex inheritance patterns have been discovered. In this study, we identified a compound heterozygous variant in *NOBOX,* a previous dominant pathogenic gene, and digenic heterozygous variants in *MSH4* and *MSH5* that had never been reported in POI. Our findings provided new insights into the complexity of POI genetics.

NOBOX is an oocyte-specific transcriptional factor that plays a critical role in early folliculogenesis. Heterozygous *NOBOX* variants can explain up to 6.2% of POI patients via a dominant negative effect or haploinsufficiency [33]. The mutation prevalence of *NOBOX* in our cohort was 1.2% (6/500). Additionally, one compound heterozygous mutation p.R355H and p.L558fs was found in two POI patients in pedigree F254. The variant p.R355H was proved to disrupt the transcriptional function of NOBOX [10], while the variant p.L558fs results in a truncated NOBOX protein lacking the C-terminal 133 amino acids. However, the proband’s mother with p.R355H mutation presented with normal menstruation cycles and menopause occurred at 48 years of age. It might be explained by an incomplete penetrance effect of the causal variant. Therefore, more evidence is needed to prove the pathogenicity of heterozygous variants in *NOBOX* for POI.

Genes involved in meiosis are critical for early follicular development. To date, majority of mutations in meiotic genes have been found in biallelic state (homozygous or compound heterozygous), such as *HFM1*, *BRCA2*, and *STAG3* [7]. *MSH4* and *MSH5* belong to the DNA mismatch repair gene family. The MSH4-MSH5 heterodimer plays an important role in homologous recombination repair of DNA double strand breaks, which is essential for meiosis [3]. WES in POI pedigrees has identified two homozygous variants in *MSH4* and *MSH5* previously [34, 35]; however, the contribution of *MSH4* or *MSH5* variants in the pathogenesis of sporadic POI has not been reported yet. In the present study, one homozygous variant in *MSH5* and three compound heterozygous variants in *MSH4* inherited in recessive pattern were identified in 5 patients, accounting for 1.0% (5/500) of patients with sporadic POI. Interestingly, patient POI-9 carried digenic heterozygous variants in *MSH4* and *MSH5*, indicating that not only one subunit deficiency, but also dysfunctional MSH4-MSH5 interaction or cumulative haploinsufficiency of both subunits, may disrupt homologous recombination during meiosis, finally causing POI. This is the first report about digenic heterozygous variants occurred in MSH4-MSH5 heterodimer, which sheds new light on the complex genetic architecture of POI and suggests a novel mechanism of POI pathogenesis.

### Digenic or multigenic variants affect the severity of POI phenotype

Previous NGS studies showed that 36%-42% of POI patients carried two or more variants in distinct genes [6, 7]. It is speculated that accumulated genetic defects or deleterious environmental exposures might aggravate the insufficient formation or accelerate the exhaustion of oocytes, resulting in diverse severity of POI phenotype. In general, menarche occurs depends on a maturing hypothalamic-pituitary-ovarian (HPO) axis. The insufficient ovarian function presents with delayed or diminished response to pituitary hormones. In this study, compared to the patients with monogenic variants, the 9 patients (1.8%) carrying digenic or multigenic variants tended to exhibit delayed age at menarche, earlier age of POI onset, and greater prevalence of primary amenorrhea. However, the above differences did not reach statistical significance, which may be due to the limitation of small sample size. Similarly, a recent study suggested that the most severe phenotypes were associated with either the major number of variations or a worse prediction in pathogenicity of variants [8]. To a certain extent, our results partially indicated that oligogenic should be considered when affected women in a family present with different phenotypes or diverse severities of POI.

One of the strengths of the present study is the largest cohort of POI patients included. Another strength is that all the candidate genes have reported evidence of confirmative pathogenicity to human POI, and that the criteria for pathogenic and likely pathogenic used to define causative variants is more strict, which is also the possible explanation for relatively lower variant frequency compared to previous studies (14.4% vs. 19% ~ 48%). There were also a few limitations. First, although the identified variants were checked in the public population data, sequencing of control women from Chinese descent is lacking. Second, the coverage of this panel did not capture all known POI-related genes. Finally, not all family members were available for co-segregation analysis or tracing variants initiation.

## Conclusions

In conclusion, a self-designed targeted gene panel covering 28 causative genes of POI used in 500 patients expanded the variant spectrum and genetic architecture of POI. Specific variants in pleiotropic genes may result in isolated POI; whereas oligogenic defects could exert cumulative deleterious effect on severity of POI phenotype. A fuller understanding of POI genetics would contribute to the individualized prediction, diagnosis, and intervention for women with POI or at high risk of developing POI.

## Methods

### Patients

A cohort of women with POI were recruited form the Reproductive Hospital Affiliated to Shandong University from 2006. Five hundred Chinese Han patients with non-syndromic POI were selected from that cohort. All patients suffered oligomenorrhea or menopause before 40 years of age and presented with elevated FSH (> 25 IU/L) at least twice over an interval of one month. Women with chromosomal abnormalities, ovarian surgery, chemo/radiotherapy, or known autoimmune disease (such as systemic lupus erythematosus, Sjögren syndrome, rheumatoid arthritis, autoimmune thyroiditis, and so on) were excluded. The peripheral blood samples were collected at the time of enrollment. All participants signed informed consent forms. The clinical characteristics of the participants were shown in Table 2.

**Table 2:** Clinical characteristics of 500 patients with POI

Characteristic	500 POI patients (mean ± SD)
Age (29)	29.72 ± 4.36
FSH* (IU/L)	69.10 ± 31.79
E2* (pg/ml)	21.33 ± 16.73
BMI (kg/m^2^)	21.37 ± 6.64
Age at menarche (29)	14.62 ± 3.04
Onset age of POI (29)	23.97 ± 5.71
Karyotype ^b^	46, XX
PA/SA (%) ^a^	22.7%/77.3%

^*^ Hormone levels are the basis levels tested during menstruation

^a^*PA* Primary amenorrhea, *SA* Secondary amenorrhea

^b^ All the patients have been tested their karyotype

### NGS and bioinformatics analysis

Based on the mutation frequencies identified previously [3, 18, 36], 28 causative genes with confirmative functional evidence were included in the target panel, including 11 meiosis genes (*HFM1, MSH4, MSH5, SPIDR, SMC1B, SYCE1, STAG3, MCM8, MCM9, NUP107* and *CSB-PGBD3)*, 8 ligands and receptors associated genes (*AMH*, *AMHR2*, *BMP15*, *BMPR2*, *FSHR*, *GDF9*, *PGRMC1* and *KHDRBS1*) and 9 transcription factors preferentially expressed in the ovaries (*FIGLA*, *FOXL2*, *NOBOX*, *NR5A1*, *POLR2C*, *SOHLH1*, *WT1*, *NANOS3*, and *LHX8*) (Table 3). The selected genes satisfied at least one of the following two requirements: 1) pathogenic mutations of the gene has been identified in women with POI; 2) functional studies have been performed to confirm that the genes involved in ovarian function maintenance. Genomic DNA was extracted from peripheral blood, and the sequencing library was prepared using the Ion AmpliSeq Library Kit 2.0 (ThermoFisher Scientific, USA). The prepared library was sequenced on MiSeqDx (Illumina, USA) using the MiSeqDx Universal Kit V3 SBS (Illumina, USA) according to the standard protocol supplied by Illumina. Majority of the variants identified by the panel testing were single-base substitutions and micro-insertions or deletions. We referred to all nonsynonymous variants, frameshift variants, and variants affecting splicing as protein-truncating variants that might affect the function of candidate genes. Rare variants with an incidence below 0.1% in the 1000 Genomes and gnomAD databases were analyzed further.

**Table 3:** The list of POI genes included in the panel

Classification	Genes	Gene OMIM number	Mode of inheritance^a^	OMIM phenotype
**Ligands and receptors associated genes**	*AMH*	600,957	AD	Persistent Mullerian duct syndrome type I
	*AMHR2*	600,956	AD	Persistent Mullerian duct syndrome type II
	*BMP15*	300,247	XLD	Ovarian dysgenesis 2; Premature ovarian failure 4
	*BMPR2*	600,799	AR/AD	Pulmonary hypertension 1; Pulmonary venoocclusive disease 1
	*FSHR*	136,435	AR/AD	Ovarian dysgenesis 1; Ovarian hyperstimulation syndrome; Ovarian response to FSH stimulation
	*GDF9*	601,918	AR	Premature ovarian failure 4
	*PGRMC1*	300,435	XLD	/
	*KHDRBS1*	602,489	AD	/
**Meiosis genes**	*ERCC6-PGBD3*	609,413	AD	Premature ovarian failure 11
	*HFM1*	615,684	AR	Premature ovarian failure 9
	*MSH4*	602,105	AR	/
	*MSH5*	603,382	AR	Premature ovarian failure 13
	*SPIDR*	615,384	AR	/
	*SMC1B*	608,685	AR/AD	/
	*SYCE1*	611,486	AR	Premature ovarian failure 12; Spermatogenic failure 15
	*STAG3*	608,489	AR	Premature ovarian failure 8
	*MCM8*	608,187	AR	Premature ovarian failure 10
	*MCM9*	610,098	AR	Ovarian dysgenesis 4
	*NUP107*	607,617	AR	Ovarian dysgenesis 6
**Transcription factors**	*FIGLA*	608,697	AD	Premature ovarian failure 6
	*FOXL2*	605,597	AD	Blepharophimosis, epicanthus inversus, and ptosis; Premature ovarian failure 3
	*NOBOX*	610,934	AD	Premature ovarian failure 5
	*NR5A1*	184,757	AD	Premature ovarian failure 7
	*POLR2C*	180,663	AD	/
	*SOHLH1*	610,224	AR/AD	Ovarian dysgenesis 5
	*WT1*	607,102	AD	Denys-Drash syndrome; Frasier syndrome
	*NANOS3*	608,229	AR	/
	*LHX8*	604,425	AR/AD	/

^a^ *AD* Autosomal dominant inheritance, *AR* Autosomal recessive inheritance, *XLD* X-linked dominant inheritance

The potential variants were classified as pathogenic (P), likely pathogenic (LP), and variants of uncertain significance (VUS) according to the guidelines proposed by the American College of Medical Genetics and Genomics [9, 37]. Pathogenic variants referred to nonsense or frameshift variants, variants located in the canonical splice site (≤ 2 intronic base pairs from the intron/exon boundary), and those previously reported to be pathogenic. Likely pathogenic variants referred to non-synonymous missense variants with a bioinformatics pathogenicity prediction of “Deleterious” by metaSVM combined with a CADD score > 3 or a DANN score > 0.95 [38, 39]. An overview of the filtering process was shown in Fig. 1.

### Sanger sequencing and haplotype analysis

The filtered variants were confirmed by Sanger sequencing. The parents of POI patients carrying compound heterozygous variants or multigenic variants were also sequenced when their DNAs were available. The primers were designed by Primer Premier 5.0 (Premier Biosoft, USA). PCR products were analyzed by agarose gel electrophoresis and purified by oligogenic glycol precipitation and then sequenced on an ABI 3730XL DNA analyzer (Applied Biosystems, Forster City, CA) using the ABI-Prism Big-Dye Terminator Cycle Sequencing Ready Reaction Kit (Applied Biosystems). Sequencing data were analyzed using the Sequencher 4.9 software (Gene Codes Corporation, USA).

### Plasmids construction and dual-luciferase reporter assay

The human coding sequence of the *FOXL2* gene was cloned into pcDNA3.1 vector with double restriction enzyme *BamHI* and *XhoI*. The mutant plasmids carrying *FOXL2* p.R349G were generated by point mutation strategy using the wild-type plasmids as template. The luciferase reporter plasmids were purchased from GeneCopoeia (http://www.igenebio.com/), termed as pProDuoLuci-*CYP11A1* (product ID HPRM30715), pProDuoLuci-*CYP17A1* (product ID (product ID HPRM30087) and pProDuoLuci-*CYP19A1* (product ID HPRM30088). All constructs were validated by Sanger sequencing.

Human embryonic kidney (HEK) 293 cells were cultured in Dulbecco’s modified Eagle’s medium (DMEM) containing 10% fetal bovine serum (FBS). The cells were plated in 24-well plates and transfected using Lipofectamine™ 3000 reagent (Invitrogen, Carlsbad, CA, USA). The total DNA concentration in each well was maintained at 500 ng, including the *CYP11A1, CYP17A1 or CYP19A1* promoter double-luciferase reporter plasmids 250 ng/well, and wild-type and/or mutant *FOXL2* or empty pCDNA3.1 vector 250 ng/well). After transfection for 48 h, luciferase activities were assessed via the dual-luciferase reporter assay system (Promega). Results were normalized against Renilla luciferase activity. The experiments were repeated at least three times.

### Statistical analysis

SPSS v.25 software was used for the statistical analysis. Data were analyzed using one-way ANOVA tests and chi-square tests, and *p*-values < 0.05 were considered statistically significant. Data were shown as means ± SD.

## Data Availability

The variations data in the study have been submitted to ClinVar data base (https://www.ncbi.nlm.nih.gov/clinvar/) under the project accession number SCV001877109—SCV001877169. The authors affirm that all data needed to confirm the conclusions of the study are present in the current article.
